# Treatment of superimposed preeclampsia on chronic hypertension in a twin pregnancy with automatic continuous positive airway pressure: a case report

**DOI:** 10.1186/s12890-020-01196-6

**Published:** 2020-06-03

**Authors:** Mi Sun Kim, Myoung Jin Moon, Yoon Hee Lee, Kyu Young Chae, Eun Hee Ahn

**Affiliations:** 1 Department of Obstetrics and Gynecology, CHA Bundang Medical Center, 59 Yatap-ro, Seongnam, 13496 Republic of Korea; 2 Department of Pediatrics, CHA Bundang Medical Center, 59 Yatap-ro, Seongnam, 13496 Republic of Korea

**Keywords:** Obstructive sleep apnoea, Hypertensive disorders of pregnancy, Continuous positive airway pressure, Twin pregnancy

## Abstract

**Background:**

Obstructive sleep apnoea (OSA) is related with adverse pregnancy outcomes, including preeclampsia. However, there are small studies about treatment of OSA with automatic continuous positive airway pressure (CPAP) in adverse obstetric outcomes.

**Case presentation:**

We introduce a case of 34 year old twin pregnant woman diagnosed with superimposed preeclampsia on chronic hypertension at 28 + 1/7 weeks of gestation. A level III polysomnography showed obstructive sleep apnoea, and automatic CPAP was applied. After the CPAP treatment concomitant with an antihypertensive drugs, both blood pressure and urinary protein concentration were reduced. The pregnancy safely continued for 49 days (to 35 + 1/7 weeks), with stable blood pressure, allowing prolongation of gestation of the foetuses.

**Conclusion:**

This is the first case to report OSA with preeclampsia in a twin pregnancy. Our results suggest that automatic CPAP as an adjunct treatment to antihypertensive drugs may be beneficial in controlling blood pressure in early-onset preeclampsia associated with OSA.

## Background

Obstructive sleep apnoea (OSA) is a common type of sleep disorder resulting from repetitive narrowing and collapsing of the upper airway; its prevalence is estimated to be 15–20% among obese pregnant women [1–3]. OSA causes daytime drowsiness and impairs daily functioning, thereby reducing the quality of life and contributing to cardiovascular diseases such as systemic hypertension, coronary artery disease, stroke, and atrial fibrillation [4, 5].

The aetiology and mechanism of OSA is multifactorial but is largely due to the interaction of an easily collapsible upper airway with the relaxation of the pharyngeal dilator muscles [6]. Obesity, advancement of age, alcohol intake, smoking, soft tissue hypertrophy, and craniofacial features such as retrognathia add to this tendency for collapse by increasing extraluminal tissue pressures surrounding the upper airway. Additionally, specific physiological changes such as weight gain, increased fluid retention, increased mucosal oedema, and hormonal homeostasis in pregnancy can contribute to the possibility of developing OSA [4, 6–8].

Pregnant women, especially those with chronic hypertension, gestational diabetes, history of preeclampsia, and/or twin gestation are at an increased risk for developing OSA, and the incidence of OSA increases throughout the trimester of pregnancy in these women [8, 9]. There are no specific guidelines for the treatment of OSA in pregnancy [7]. However, some studies have investigated the possible role of continuous positive airway pressure (CPAP) as an adjunct treatment for pregnant women who are at a risk of preeclampsia [10, 11].

The case presented here is a woman with a twin pregnancy who was diagnosed with superimposed preeclampsia on chronic hypertension and OSA, in whom CPAP treatment along with an antihypertensive drug resulted in good control of blood pressure (BP) and prolongation of the pregnancy.

## Case presentation

A 34-year-old, primigravid woman presented at 28 + 1/7 weeks of gestation. She conceived dichorionic diamniotic twins following in vitro fertilization via an intracytoplasmic sperm injection in the setting of severe adenomyosis. Routine pregnancy examinations were performed from early gestation, and clinical findings were normal. She was diagnosed with hypertension 2 years prior to the pregnancy and was treated with 30 mg nifedipine twice daily. Her blood pressure was controllable during first and second trimester; systolic (120-140 mmHg) and diastolic (70-90 mmHg). She was admitted to our department due to superimposed preeclampsia on chronic hypertension and preterm labour.

At admission, she had a BP of 160/100 mmHg and urinary protein concentration of 2.5 g/L. No headache, epigastric pain, visual disturbance, or hyperreflexia were complained. Her platelet count and liver enzyme levels were normal. An ultrasound scan showed appropriate-for-gestational-age foetuses (estimated foetal weight of the 15th and 50th centiles), with normal amniotic fluid volume.

**Fig. 1:**
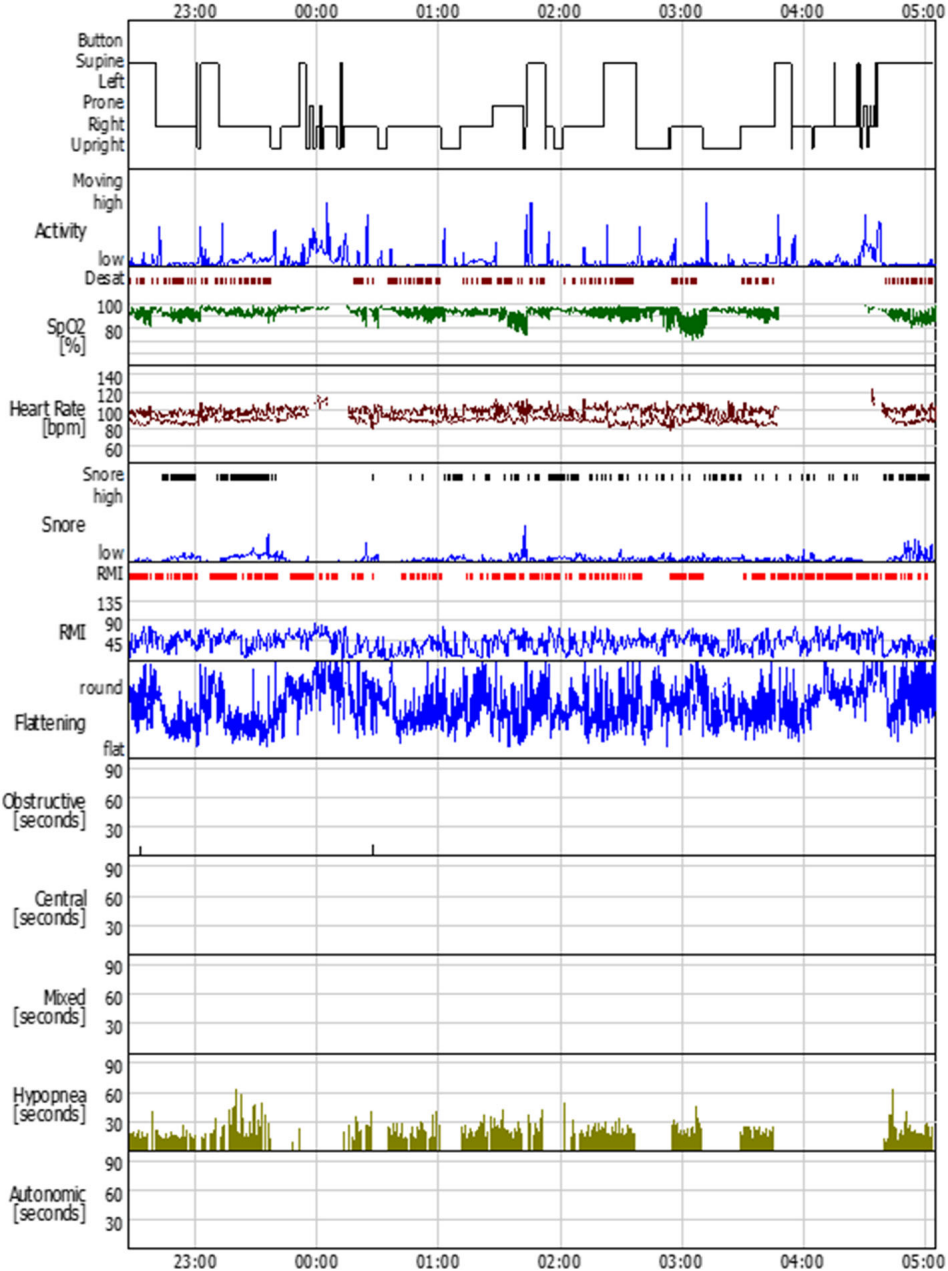
Level III polysomnography (PSG). PSG confirmed the presence of severe obstructive sleep apnoea (OSA) with an apnoea–hypopnea index of 67.5 and severe airflow limitation

**Fig. 2:**
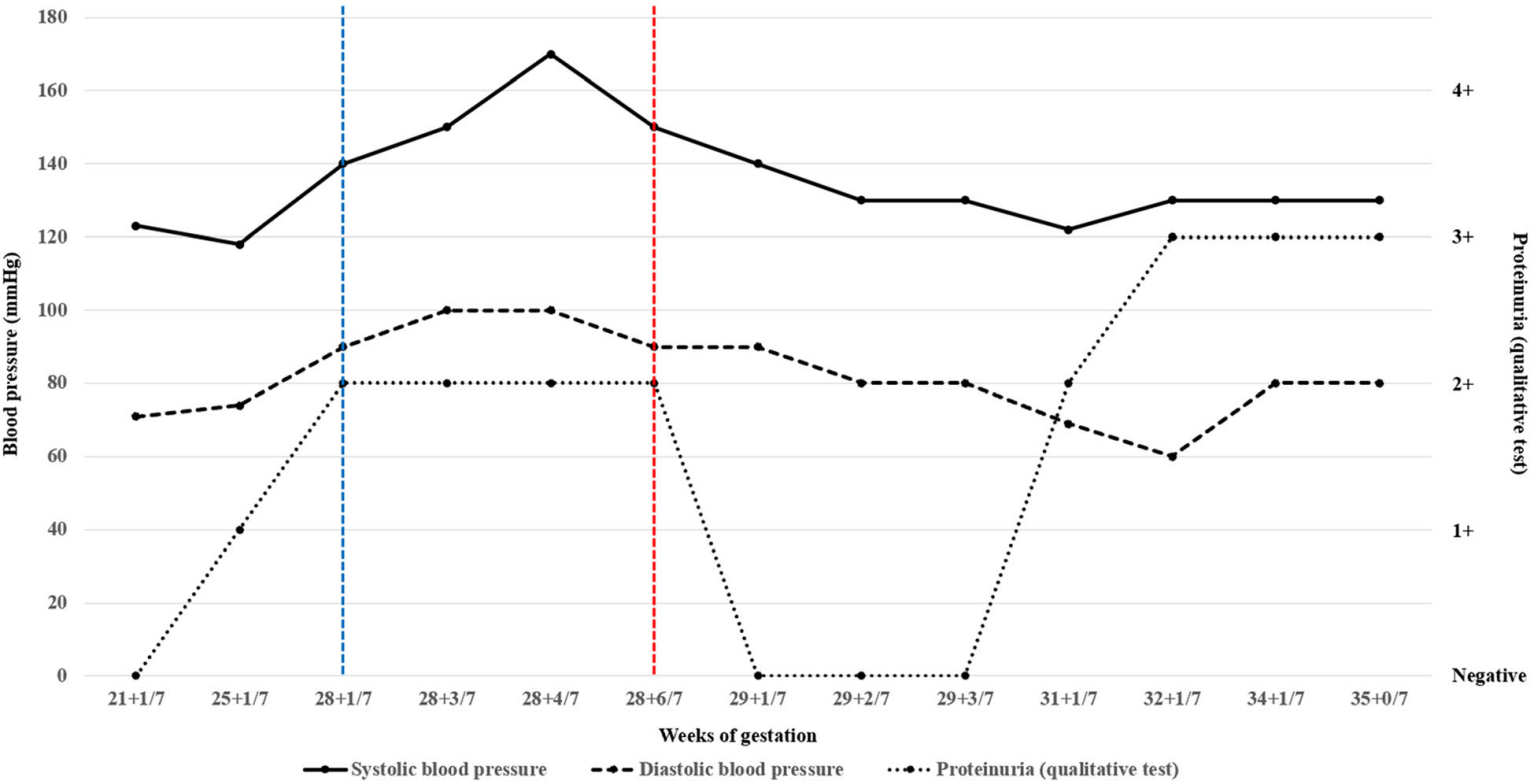
Clinical markers of preeclampsia. The clinical markers of superimposed preeclampsia improved after the start of the automatic continuous positive airway pressure (CPAP) treatment. Systolic and diastolic blood pressures normalized with CPAP treatment until delivery. No proteinuria was found for 2 weeks after the initiation of CPAP. The blue line represents the admission day and the red line represents the start of CPAP treatment

Throughout her hospital stay, hypertension was stabilized with hydralazine 15 mg. The use of nifedipine was continued till admission and then stopped in order to allow the safe use of magnesium sulfate as an anticonvulsant. During sleep, she snored loudly. Repeated drop in peripheral oxygen saturation to a nominal value of 70% was revealed in pulse oximetry. She told that she felt tired during daytime and would wake up every hour during the night. Physical examination revealed a body mass index of 40 kg/m^2^, a large neck, and a low soft palate. Level III polysomnography (PSG) confirmed the presence of severe obstructive sleep apnoea (OSA) with an apnoea–hypopnea index (AHI) of 67.5 (number of apnoeic plus hypopneic episodes per hour; normal range, < 5) and severe airflow limitation (Fig. 1). The nadir oxygen desaturation was at 71.0%. The oxygen saturation dropped below 90 and 80% for 20.2 and 2.5% of the total sleep time, respectively. Treatment with nocturnal nasal automatic CPAP at 8–12 cmH_2_O resulted in a significant reduction in the peripheral desaturation and BP. After receiving CPAP, she didn’t require any hypertensive drug such as hydralazine until discharge. She was discharged after 4 days of the CPAP treatment with a BP of 130/80 mmHg and an absence of protein in urine analysis. After discharge, she continued to take 30 mg nifedipine twice daily as usual with CPAP use at night, and her BP remained stable throughout the pregnancy (Fig. 2).

The mother proceeded to have a caesarean section at 35 weeks of gestation due to uncontrolled preterm labour and transverse-lie foetal presentation. Both her babies were healthy, with birth weights of 1700 g (5th percentile by normogram in twin gestation) and 2335 g (40th percentile). Apgar scores were 5 and 9 for infant one and 8 and 9 for infant two, at 1 and 5 min respectively.

## Discussion and conclusions

To our knowledge, this is the first case report of a hypertensive disorder in pregnancy (HDP) and OSA in a twin pregnancy in which BP was well controlled and prolongation of pregnancy resulted following automatic CPAP as an adjunct treatment to an antihypertensive drug.

The prevalence of OSA in pregnancy with an AHI of greater than 5 has been reported to be 3.6% in the first trimester and 8.3% in the second and third trimesters [7]. Throughout the pregnancy OSA exacerbates and is connected to poor maternal and foetal outcomes [4, 12]. Pregnant women with habitual snoring, unexplained hypoxemia, chronic hypertension, and high pre-pregnancy BMI strongly benefits from evaluation for underlying OSA and can be referred to a sleep medicine specialist for evaluation and management [1, 7, 13]. Data are lacking concerning the best timing of screening for OSA in prenatal visits. The gold standard for the diagnosis of OSA in pregnancy is an overnight, attended, PSG [7].

The mechanisms that link HDP to OSA are not clear, but several pathways are possible. The pathophysiologic OSA may be related to HDP in this following ways: airflow restriction and awakening from sleep and intermittent hypoxemia. These phenomena, combined with intrathoracic pressure variations, trigger chemoreflexes that stimulate the sympathetic drive and lead to inflammation, vasoconstriction, and vascular endothelial dysfunction that predispose to hypertension [3, 12, 14]. Edward et al. found that BP increases following upper airway occlusion in 10 pregnant women with OSA combined with preeclampsia compared to 10 women with OSA only [15].

Associations between OSA and chronic hypertension have been reviewed in the literature [1–3, 13]. Chronic hypertension was more common in pregnant women with OSA in a prospective study of over 3000 women and the incidence of chronic hypertension rises with OSA severity [3]. O’Brien et al. reported that the incidence of OSA in chronic hypertension was higher (43%) versus the normotensive controls (19%) in pregnant women. Chronic hypertension was more frequent in those with chronic snoring, and gestational hypertension was more common in those who reported new onset of snoring in current pregnancy [13].

The American College of Physicians guideline recommends that all overweight and obese patients diagnosed with OSA should be encouraged to lose weight, go through CPAP treatment, or use a mandibular advancement device as an alternative therapy to CPAP [16]. Automatic CPAP, which was used in this case, has been promoted as having an added benefit over CPAP [17]. The devices respond to respiratory flow, flattening of the inspiratory flow contour, snoring, generator speed, or impedance of the upper airway. It is designed to adjust the treatment pressure to the actual requirement of the patient, improving patient compliance and acceptance [18]. Both the devices prevent upper airway obstruction in all sleep stages and body positions in order to restore normal sleep [18].

No general guidelines for the treatment of OSA in pregnancy is available [7]. In general population weight loss is recommended; however, this is not recommended during pregnancy. Achieving standard weight before pregnancy is essential. Studies have reported the possible role of CPAP in pregnant women who are at risk of HDP as an adjunct treatment, but there is still a lack of evidence for its efficacy [10, 11]. The goal of CPAP therapy is to control the apnoea hypopnea index (AHI) to less than 5 and prevent recurrent oxygen desaturation [1, 7]. In preeclamptic women, CPAP may have a part in improving BP and increasing cardiac output during sleep [10, 19]. In a study of 11 preeclampsia women, nocturnal BP rise was effectively removed by the application of CPAP [15]. Furthermore, Blyton et al. demonstrated that CPAP treatment minimized cardiac output reduction and minimized increase in total peripheral resistance during sleep in pregnancy with preeclampsia [10]. Similar to our case, Whitehead et al. described a case of untreated OSA with severe preeclampsia that was managed with night CPAP. Improvement of BP with no medication, a decrease in urinary protein was shown by the CPAP therapy. Furthermore, the physicians were able to delay labour induction for a month after the diagnosis of severe preeclampsia [20]. In contrast, J Reid et al. demonstrated no significant improvement in BP or inflammatory markers in 24 women randomly assigned for either CPAP or a mandibular advancement device with a nasal dilator strip [11]. Based on current prospective observational studies, CPAP has shown a beneficial effect on BP control; however, extra studies are needed to determine if CPAP can have a therapeutic benefit in the management of HDP [19–21].

Studies assessing the benefit of maternal OSA on foetal outcomes by using PSG are more incomplete than those evaluating maternal outcomes. OSA may contribute to reduced placental tissue perfusion and impairs foetal growth [12, 22]. Two prospective studies have demonstrated an association of maternal OSA with impaired foetal growth. One study reported that impaired foetal growth (defined as a birth percentile <10th centile or a fall in customized centile by > 33% between 32 weeks and term) was observed in 43% (6/14) of cases of OSA versus 11% (3/27) of controls after adjusting for BMI [22]. Another study reported that maternal OSA in the third trimester was related to delivery of small-for-gestational age infants [13].

In conclusion, to our knowledge, this is the first case of HDP and OSA in a twin pregnancy where BP was well controlled and pregnancy prolongation resulted following automatic CPAP treatment as an adjunct treatment to an antihypertensive drug. The importance of HDP and association between OSA and HDP are increasing. However, there are small studies about treatment of OSA in adverse outcomes of HDP in pregnant women. Thus, large, randomized controlled trials are needed to evaluate the impact of OSA treatment on maternal and foetal outcomes in HDP.

## Data Availability

Data sharing is not applicable to this article as no datasets were generated or analysed during the current study.

## Abbreviations

AHI: Apnoea-hypopnea index
BMI: Body mass index
BP: Blood pressure
CPAP: Continuous positive airway pressure
HDP: Hypertensive disorders in pregnancy
OSA: Obstructive sleep apnoea
PSG: Polysomnography
